# Non‐Native Rodents Dominate Understory Frugivory in the Eastern Caribbean Islands

**DOI:** 10.1002/ece3.74116

**Published:** 2026-07-31

**Authors:** Seokmin Kim, Fabio L. Tarazona‐Tubens, Maximilian G. R. Vollstädt, Fernando Gonçalves, Emmeli Agerskov Claré, Andreas Krogh Norrild, Hanna Welzel, Tianying Zhang, Mark Hulme, Christopher Kaiser‐Bunbury, Mauro Galetti, Benno Simmons, Bo Dalsgaard, Christopher Searcy

**Affiliations:** ^1^ Department of Biology University of Miami Coral Gables Florida USA; ^2^ Section for Molecular Ecology and Evolution, Globe Institute University of Copenhagen Copenhagen Denmark; ^3^ Instituto Mediterráneo de Estudios Avanzados (CSIC‐UIB) Mallorca Balearic Islands Spain; ^4^ Department of Evolutionary Biology and Environmental Studies University of Zurich Zurich Switzerland; ^5^ Department of Life Sciences The University of the West Indies St. Augustine Trinidad and Tobago; ^6^ Centre for Ecology and Conservation, Faculty of Environment, Science and Economy University of Exeter Penryn UK; ^7^ Department of Biodiversity São Paulo State University (UNESP) Rio Claro Sao Paulo Brazil

**Keywords:** biogeography, biological invasion, birds, rodents, species interactions

## Abstract

Biotic invasions represent one of the leading environmental threats, with non‐native mammals being particularly damaging to native biodiversity on oceanic islands. One way that such damages may occur is through the disruption of frugivory, a fundamental process for plant reproduction and healthy ecosystem function. To investigate such disruptions, we explored (1) the magnitude of non‐native mammalian frugivore activity, (2) whether it affects native frugivory interaction rates, and (3) which local, biotic, biogeographic, and socio‐economic factors best predict non‐native mammal frugivory levels. We used artificial fruits and camera traps to measure native and non‐native frugivory interactions on 13 islands in the eastern Caribbean, an important biological hotspot. Overall, we found that non‐native *Rattus* sp. were responsible for nearly 80% of all frugivorous activity detected by camera traps and that increased non‐native mammalian frugivore activity was associated with significant declines in avian frugivory rates. Non‐native mammalian frugivore activity, in turn, was positively associated with road proximity and neighboring island proximity, illustrating the potential for both human activity and island geography to influence non‐native mammal frugivory interactions. These results suggest that non‐native mammals are dominating frugivory dynamics in understory vegetation in the eastern Caribbean, with consequences for native frugivory interactions. Given the generally seed predatory behavior of *Rattus* sp., we argue that plant dispersal could be negatively affected on islands with abundant non‐native mammal populations. Thus, we emphasize the need for controlling non‐native mammal populations and highlight the diverse inter‐trophic effects that non‐native mammals can have on insular tropical ecosystems.

## Graphical abstract

We investigated how non‐native mammals have disrupted frugivory by using artificial fruits and camera traps to measure native and non‐native frugivory interactions on 13 islands in the eastern Caribbean. We found that non‐native *Rattus* sp. were responsible for nearly 80% of all frugivorous activity and that increased non‐native mammal activity was associated with significant declines in avian frugivory rates. These results suggest that non‐native mammals are dominating frugivory dynamics in understory vegetation in the eastern Caribbean, with consequences for native frugivory interactions.

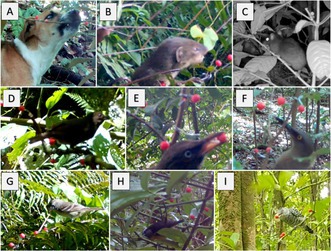

## 1 Introduction

Biological invasions represent one of the most damaging components of anthropogenic global change, being a major driver of extinctions and declines in species richness (Blackburn et al. 2004; Mollot et al. 2017; Haji et al. 2023), the displacement of native species from their habitats (Huxel 1999), and the disturbance of ecological processes (Hicke et al. 2012; Gonçalves et al. 2020). One prominent ecological process that has been altered by species invasions is plant‐frugivore interactions (García et al. 2014; Vollstädt et al. 2022; Zhu et al. 2023), which can have negative implications for the maintenance of community composition and diversity of plants and their frugivores (Traveset and Richardson 2006). The colonization success of non‐native animals is often linked to their generalized diets, which allows such species to exploit a broad range of resources (Stigall 2012; Haji et al. 2023). While it is possible for non‐native species to replace many native frugivore functions (Peris et al. 2019; Vizentin‐Bugoni et al. 2019), some functions are lost as novel plant‐frugivore interactions may not fully replace the roles of native seed dispersers (Heinen et al. 2023). Therefore, non‐native frugivores can render affected ecosystems more susceptible to further anthropogenic disturbances (Neuschulz et al. 2016).

Among non‐native frugivores, non‐native mammals have been suggested to be particularly damaging to plant‐frugivore interactions. Driven by invasive rodents with broad diets such as rats of the genus *Rattus*, these invasions have led to significant increases in seed mortality due to granivory (Dammhahn et al. 2017; Miller‐ter Kuile et al. 2021). Additionally, non‐native mammals could suppress native frugivores, particularly birds, through predation and resource competition (Wilson Rankin et al. 2018; Nance et al. 2023), with potentially damaging effects on plant communities through the disruption of native mutualistic interactions (Heinen et al. 2023). Given the global scope of non‐native mammal introductions due to human‐mediated transportation (Genovesi 2010), these invasions have wide‐reaching consequences for a variety of ecosystems across the globe, especially island ecosystems (Russell and Kueffer 2019). As species on oceanic islands have evolved largely without mammalian predators, insularity has caused many island species to lose defensive traits, making these species vulnerable to non‐native mammals (Blackburn et al. 2004; Doherty et al. 2016). Islands, particularly tropical islands, support a disproportionately high level of global biodiversity (Kier et al. 2009). However, the same insularity that promotes endemism also increases vulnerability to extinction. For instance, Schrader et al. (2024) found that 51% of all threatened vascular plants occur on islands, the majority of which are large‐fruited species that now face severe dispersal limitations due to anthropogenic extinctions and mutualistic disruption (Tarazona‐Tubens et al. 2026). Meanwhile, Matthews et al. (2022) revealed that 80% of all bird extinctions over the past 125,000 years were island endemics. Therefore, it is of high priority to protect insular biodiversity hotspots from further anthropogenic disturbances and degradation caused by invasive species. Consequently, understanding the effects of ongoing non‐native mammal invasions on important ecosystem functions (Goedert et al. 2020; Cooper 2008; Kaiser‐Bunbury et al. 2010), such as plant‐frugivore dynamics, could provide important insights into the ecological effects of non‐native mammalian invasions on islands.

In this study, we aim to broaden our understanding of the effects of non‐native mammals on plant‐frugivore interactions in tropical insular ecosystems by using the islands in the eastern Caribbean (part of the larger Caribbean biodiversity hotspot) as a study system (Myers et al. 2000). Given the variation in area, levels of isolation, age, and geological origin (Maunder et al. 2008), and their vulnerability to anthropogenic disturbances such as habitat loss and invasive species (Brooks et al. 2002; Hays and Conant 2007), these islands provide an ideal model system to examine the effects of non‐native mammals on frugivory in an island biogeographical context. We ask the following questions: (1) What is the magnitude of non‐native mammalian frugivory across the eastern Caribbean? (2) How does non‐native mammalian frugivore activity affect native plant‐frugivore interaction rates? and (3) Which biotic, biogeographic, socio‐economic, and local/site‐specific characteristics best predict non‐native mammalian frugivore activity? To investigate these questions in a standardized way, we recorded frugivory across 13 islands in the eastern Caribbean using artificial plasticine fruits and camera traps. We then tested which relevant environmental metrics (Table S1) best predict both total frugivory rates and non‐native mammalian frugivore activity. Given the high density of invasive mammals on islands (Hays and Conant 2007) and their opportunistic diets (Dammhahn et al. 2017), we expected to find a clear negative relationship between non‐native mammalian frugivore activity and native frugivory. Furthermore, we anticipated that the abundance of non‐native mammal interactions would be best explained by socio‐economic factors given the role that anthropogenic effects have on driving species invasions (Genovesi 2010).

## 2 Methods

### 2.1 Study System

**FIGURE 1:**
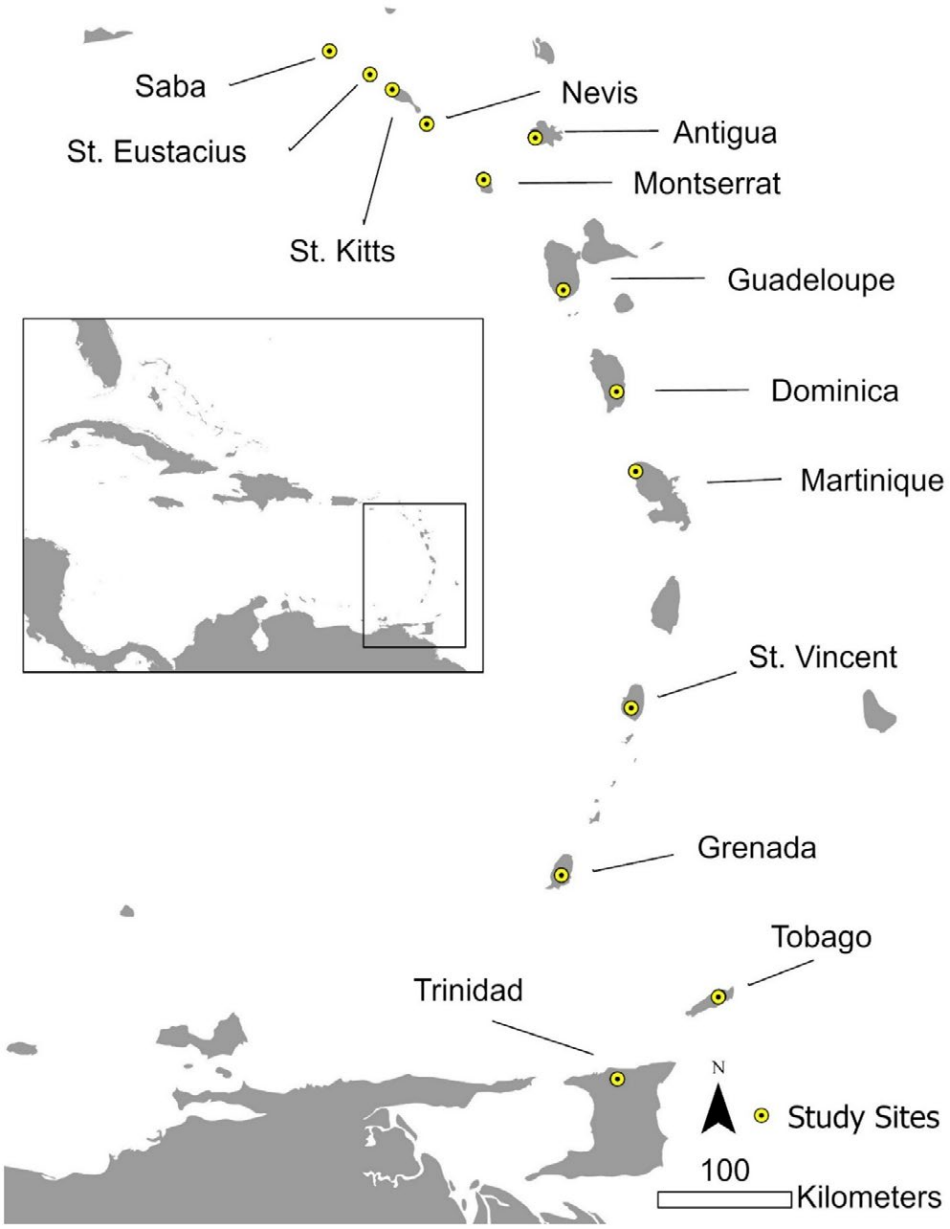
Study sites within the eastern Caribbean.

This study took place on 13 islands in the eastern Caribbean, which includes 11 Lesser Antillean islands ranging from Saba in the north to Grenada in the south and two continental islands—Trinidad and Tobago. We conducted most of this study between February and May 2023, with only Antigua and Grenada sites being surveyed in May 2022. The Lesser Antilles make up the easternmost section of the broader Caribbean region. Geologically, these islands (some of which are still volcanically active; Dalsgaard et al. 2007) are among the youngest in the Caribbean, with most of the islands being formed due to volcanic activity < 20 million years ago (Ricklefs and Bermingham 2008). The islands have relatively high elevational gradients, with five of our study islands having elevations higher than 1000 m (Table S2). Thus, these islands contain a variety of habitats including dry thickets, rain forests, seasonal forests, and high‐altitude forests. To the east of the main volcanic arc are several younger low‐lying islands, such as Antigua, consisting mostly of limestone. Although a few Lesser Antillean islands were interconnected during the Last Glacial Maximum due to low sea level (Antigua and Barbuda; St Kitts, Nevis, and St Barts; Grenada and the Grenadines; Ricklefs and Bermingham 2008), the Lesser Antillean islands have been largely isolated from continental landmasses since their formation (Thorpe et al. 2008; Figure 1). In contrast to the islands in the Lesser Antilles, Trinidad and Tobago are biogeographically associated with the South American mainland, being separated from the continent only recently (11,000 years ago and < 2 million years ago for Trinidad and Tobago, respectively; Snow 1985). Consequently, the flora and fauna on Trinidad and Tobago more closely resemble South America's and are distinct from those of the Lesser Antilles (Scott 1972; Trejo‐Torres and Ackerman 2001). However, like the Lesser Antillean islands, both islands also support a variety of habitats, ranging from dry lowlands to higher elevation rainforests (Feinsinger et al. 1982).

### 2.2 Field Methods

On each of the 13 islands in our study, we selected two approximately 1‐ha plots in which to evaluate plant‐frugivore interactions. These sites were chosen with the help of local experts based on known areas of high bird activity with representative native vegetation communities. These sites were located ~200 m apart from each other and were within forested areas at or near high elevation relative to each island (559 ± 236 m SD). To evaluate frugivory interactions, we used red modeling clay (plasticine) to form ~1 cm diameter, spherical artificial fruits (Galetti et al. 2003; Alves‐Costa and Ariadna 2001). During midday, we secured 15 of these artificial fruits using metal wires at a height of 1–2 m on 12 arbitrarily selected shrubs or small trees in the study site. Therefore, each site had 180 fruits evenly distributed across 12 plants spaced roughly 15 m apart from each other. These artificial fruits are non‐toxic and are useful for testing frugivory rates while controlling the number of fruits, their position in the environment, color, and size (Alves‐Costa and Ariadna 2001).

It is important to note that artificial fruits may not fully represent natural fruits because they only mimic fruit size, shape, and external color while lacking other cues used by frugivores, including odor, nutritional rewards, and UV reflectance (Corlett 2011). The standardized red plasticine fruits were selected to resemble the size and general appearance of many small, fleshy fruits found within our study area, but they do not replicate the diversity of fruit traits available to frugivores. Birds generally rely more heavily on visual cues, particularly color, during fruit selection (Schrader et al. 2024), whereas many mammals additionally use olfactory cues (Rodríguez et al. 2013). Likewise, willingness to investigate novel foods varies among taxa and species, potentially influencing attack rates on artificial fruits (Greggor et al. 2015). Consequently, our results should be interpreted with caution as patterns of relative interactions with standardized fruit models rather than direct measures of preferences for natural fruits. Furthermore, we only placed these fruits in the understory, thus, we may not be able to extrapolate the frugivory dynamics elsewhere in a forest, especially as both mammal and bird communities can be stratified by canopy levels (Walther 2002). Regardless of these caveats, previous research has shown that plasticine fruits can provide useful insights into plant‐frugivore dynamics (Brodie III 1993; Vasconcellos‐Neto et al. 2000; Galetti et al. 2003; Hazell et al. 2023). Moreover, our protocol allowed us to conduct the same standardized setup across all 13 islands, making our data comparable between islands.

We checked the fruits after 72 h, documenting fruit removal and frugivore marks (e.g., bird pecks and reptile bites; see Figure S1; Figure 1 in Alves‐Costa and Ariadna 2001). To help assess which animal class (e.g., mammal, bird) were responsible for fruit interactions, in addition to using frugivore marks, we set up two camera traps (Bushnell Core DS 30MP Low Glow) facing two separate plants with artificial fruits, spaced ~60 m apart per site. At the end of the 72‐h period, we recovered the camera traps, as well as any materials used for our study, and examined the camera trap footage to identify which frugivore species visited the target plants. As we were unable to place camera traps on every plant with plasticine fruits, we could not definitively determine whether each interaction was from a native or non‐native animal. For instance, camera trap footage showed both native birds and non‐native mammals fully removing artificial fruits. Thus, determining the cause of removed fruits was not possible, and was only used when calculating the overall frugivory rate rather than being partitioned between native vs. non‐native sources. In contrast, we only saw native birds interacting with artificial fruits, allowing us to consider that any artificial fruits with avian markings were interacted with only by native species. Meanwhile, camera traps were not sensitive enough to detect any lizard frugivory despite them occasionally leaving marks on the plasticine fruits.

We used two metrics of frugivory for this study: frugivory rate and frugivore activity. For frugivory rate, we considered the sum of all artificial fruits removed plus the number of artificial fruits with reptilian/avian marks for each island. For frugivore activity, we defined animal interaction footages from camera traps that were < 1 min apart as a single visit and then distinguished each visit as either mammalian or avian. Then, we averaged the number of visits between the two cameras to calculate the number of visits per site. Taking the average instead of the sum was necessary due to a limited number of camera trap malfunctions (three out of 52 deployments) during our surveys. Having these two metrics allowed us to take advantage of the large total sample size of plasticine fruits (frugivory rate) and the higher taxonomic resolution provided by the camera traps in terms of which species were interacting with the plasticine fruits (frugivore activity).

### 2.3 Environmental Characteristics

We collected environmental variables representing four broad categories: local, biogeographic, biotic, and socio‐economic. Local variables represent conditions of our specific study sites, while the other three classifications assess broader, island‐level characteristics. Each of the selected variables within these classifications have been associated with shaping plant–animal interactions and/or communities (biotic: Kissling et al. 2007; Pessoa et al. 2017; Vázquez et al. 2009; biogeographic: McFadden et al. 2022; Muñoz et al. 2019; local: Laurance 2004; Coutant et al. 2022; socio‐economic: Coutant et al. 2022; Teixido et al. 2022). While we initially considered 22 variables (8 biogeographic, 4 biotic, 5 local, 5 socio‐economic), we consolidated this list down to 13 variables (2 biogeographic, 4 biotic, 4 local, 3 socio‐economic) after appropriate transformations to optimize normality and removing highly correlated variables (cutoff: |*r*| > 0.7) (Table S2).

The final selection of local variables included site elevation, non‐native mammalian frugivore activity, avian frugivore activity, and distance to nearest road. Elevation has been linked to shaping plant‐frugivore networks, as higher elevation areas are more susceptible to disturbances due to increases in animal specialization (Quitián et al. 2018; Dalsgaard et al. 2018). Meanwhile, the distance of a site to the nearest road is often considered as a metric for the level of anthropogenic disturbance a location may experience (Delgado et al. 2007). Accordingly, distance to roads may be associated with altered frugivore behavior and plant–animal interactions (Laurance 2004). We estimated site elevation using a GPS unit (Garmin‐ GPSMAP 64sx) to mark the elevation of each of the shrubs with artificial fruits. Then for each site, we averaged the elevation between the 12 plants to calculate mean site elevation. For distance to roads, we relied on available Caribbean road and landcover GIS layers from geoMinds, a database that provides license‐free OpenStreetMap (OSM) geodata throughout the region (http://www.geominds.de/downloadcenter.html). We considered “road” as any roads that are within the primary, secondary, tertiary, trunk, or residential classifications, as these are more likely in use by vehicles than other classifications (unclassified, service, track, and path). Then, using the “Nearest Distance” tool from ArcGIS Pro 2.7.0 (2020), we measured the distance to roads from each of our sites.

Island biogeography often examines how island area, age, and isolation influence patterns of biodiversity (Simberloff and Abele 1976). These features affect fundamental biogeographic processes, such as speciation, immigration, and extinction (Whittaker et al. 2008) and may also influence mutualistic interactions (Dalsgaard and Temeles 2024). For this study, our final biogeographic variables included number of close neighboring islands and distance to nearest neighboring island. We initially considered island area, but as it was highly correlated with many variables (distance to built areas, native plant richness, length of roads, and number of tourists), it was not included in our final analysis. To determine the number of close neighboring islands, we buffered each study island by 100 km and counted the number of neighboring islands within this zone. The 100 km threshold was chosen as previous studies (Manel et al. 2019; Mellin et al. 2010; Honorio Coronado et al. 2020; Marrotte et al. 2020) considered 100 km as the distance needed for an ecosystem to be independent from other systems. For distance to nearest neighboring island, we considered the distance between the target island and either its nearest neighbor or the South American continent. We only considered islands or landmasses with areas > 20 km^2^, as GIS shapefiles for smaller islands were not readily available. These metrics were calculated through the “Buffer” and “Nearest Distance” tools from ArcGIS Pro (2020), respectively.

Biotic factors for this study included native frugivorous reptile and bird species richness, native plant species richness, and percent forest cover of each island. To estimate frugivorous reptile and bird species richness, we consulted an overview of reptile and bird species in the Caribbean (birds: Gerbracht and Levesque 2019; reptiles: Valido and Olesen 2019) and defined frugivorous species as those that consume > 10% of their diet as fruits (Kissling et al. 2007). We supplemented this survey with information from Avibase (https://avibase.bsc‐eoc.org/avibase.jsp) to form a comprehensive list of frugivorous reptiles and birds for our study area. We excluded native frugivorous mammals from our analyses as native frugivorous mammal communities for most islands in the region are largely limited to bats, and exploratory trials for this study with camera traps deployed in 2022 failed to capture any instances of bats or other native mammals interacting with artificial fruits. We selected percent forest cover and native plant richness as they represent potential fruit resource availability for frugivores. We calculated percent forest cover using forest area estimates from the most recent Sentinel‐2 Land Cover Explorer data (Karra et al. 2021) and dividing it by island area. We obtained native plant richness information from Rojas‐Sandoval et al. (2020) and Backsh‐Comeau et al. (2016).

Socio‐economic factors in this study were used to estimate the magnitude of anthropogenic effects on each island and included GDP per capita, number of international tourists, and percent built cover. Rojas‐Sandoval et al. (2020) provide information regarding number of international tourists and GDP per capita for all Lesser Antilles islands. For Trinidad and Tobago, we relied on information available from the World Bank (https://www.worldbank.org/en/home) and previous studies (Russell and Kueffer 2019; Mohan 2022). We calculated percent built cover using built area estimates from Sentinel‐2 and dividing it by island area. Built area estimates included all areas that contain human‐made structures, major road and rail networks, and large impervious surfaces such as parking lots (Karra et al. 2021).

### 2.4 Statistical Analysis

To determine how non‐native mammalian frugivore activity relates to native frugivory rates, we employed a linear mixed‐effects model using the “lme4” package in R version 4.2.1 (Bates et al. 2015; R Core Team 2024), with island identity as a random effect. Aside from non‐native mammals, our camera trap footage only showed native birds interacting with artificial fruits. Therefore, we consider that a regression between non‐native mammalian frugivore activity and the total avian frugivory rate (as measured by frequency of avian markings on artificial fruits at each site) assesses the effect that non‐native mammals have on native frugivory rates. Continental (Trinidad and Tobago) vs. oceanic (all other islands) classification was initially considered as a separate fixed effect, but as this effect was not found to be significant it was removed from the analysis. Additionally, we explored potential mechanisms behind changes in avian frugivory rates due to non‐native mammalian frugivore activity by assessing the relationship between non‐native mammalian frugivore activity and local avian abundance, species richness, and evenness through similar linear mixed effects models. Local avian information was gathered with mist nets near our study sites (< 1 km) at concurrent time periods, for a separate study on bird‐plant community dynamics. Survey effort at each site consisted of five, 12‐m long mist nets spread out linearly along a trail. These were set up between sunrise (~6 am) and noon for 5 days. This process did not occur for the islands of Guadeloupe and Martinique. Additionally, mist netting efforts and artificial fruit trials did not occur concurrently for Antigua and Grenada. Therefore, those four islands were removed from our analysis examining the relationship between non‐native mammalian frugivore activity and local avian abundance.

To evaluate which factors best predict total frugivory rates, we conducted a bidirectional elimination stepwise regression approach with total frugivory rate as the response variable and variables within each of our broad classifications as the predictors. As biotic, biogeographic, and socio‐economic characteristics were assessed at the island level, we averaged local variables (site elevation, non‐native mammalian frugivore activity, distance to nearest road) and total frugivory rates per island (*n* = 13). All models were ranked by their Akaike's Information Criterion corrected for small sample sizes (AICc), and we considered values of AICc within 2 U (ΔAICc < 2) from the top‐ranking model to have similar support (Anderson and Burnham 2002). After identifying the model most supported by the data, we evaluated the relative contribution of each individual predictor using “lmg scores” (Lindemann et al. 1980). This index is a variance decomposition metric that calculates the relative contribution of each variable to the R^2^. This procedure was calculated through the “relaimpo” package (Grömping 2006) in R (R Core Team 2024). To investigate which factors best predict non‐native mammalian frugivore activity, we followed a similar process with non‐native mammalian frugivore activity as the response variable.

## 3 Results

**FIGURE 2:**
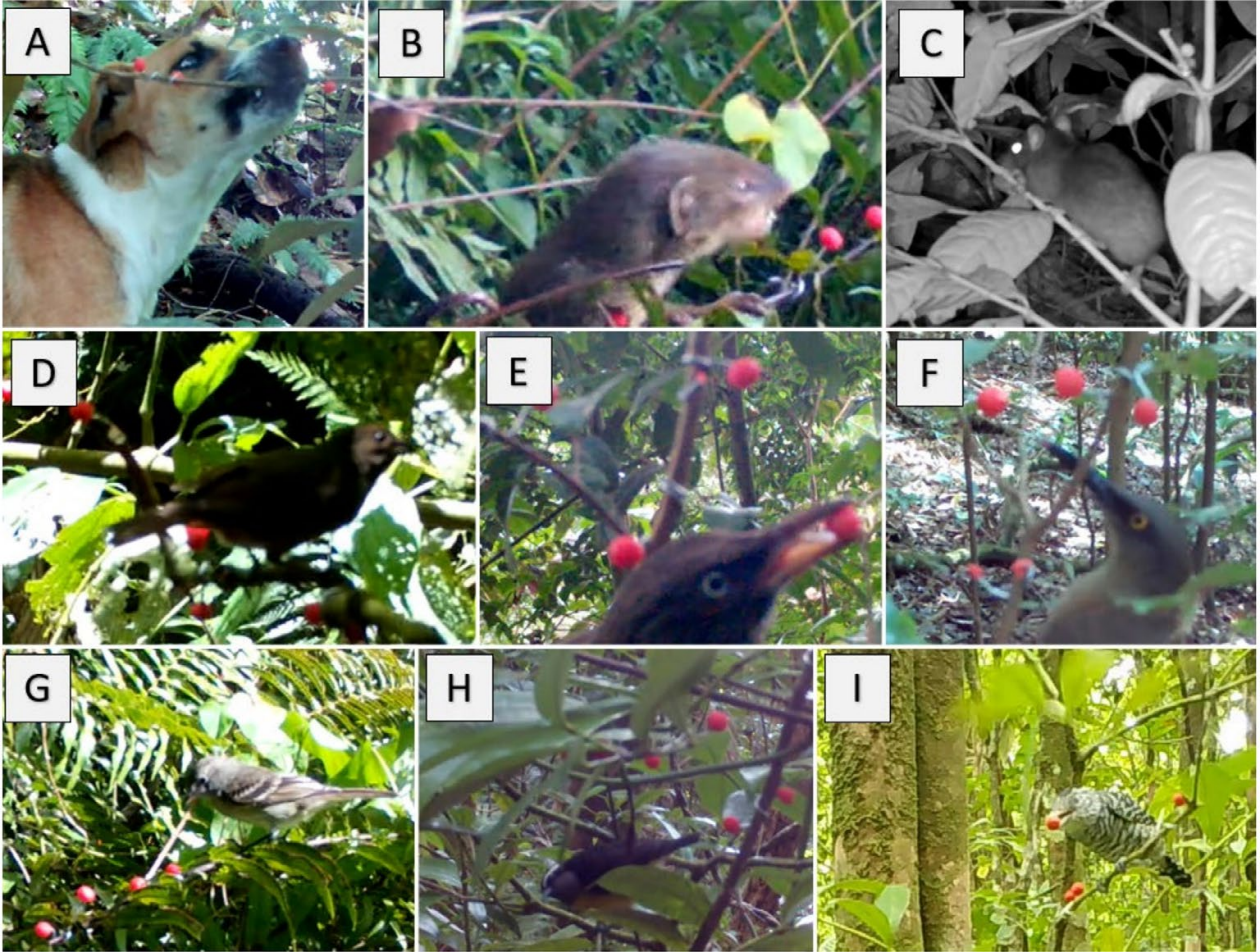
Mammalian and avian frugivores interacting with artificial fruits as detected by the camera traps in the study. (A) *Canis lupus* (common dog), (B) *Urva auropunctata* (Small Indian mongoose), (C) *Rattus* spp. (Rat), (D) *Loxigilla noctis* (Lesser Antillean bullfinch), (E) *Margarops fuscatus* (Pearly‐eyed thrasher), (F) *Cinclocerthia ruficauda* (Brown trembler), (G) *Elaenia martinica* (Caribbean elaenia), (H) *Coereba flaveola* (Bananaquit), (I) *Thamnophilus doliatus* (Barred Antshrike). Species (A–C) are non‐natives whereas (D–I) are native species.

Of the study islands, Nevis had the highest frugivory rate, with 86% (155/180) of the artificial fruits either removed or with frugivore marks. In contrast, Saba had the lowest frugivory rate, with only 16% (28/180) of the artificial fruits showing signs of frugivory. Across all islands, the most common form of interaction was complete removal of fruits (78%), followed by avian (11%) and lizard (7%) frugivory marks. Overall, our camera traps captured 61 frugivore visits. Of these, 49 (80%) were by mammalian frugivores, all non‐native, with 47 (77%) being caused by rats (*Rattus* sp.), and only a single interaction by either a dog ( *Canis lupus* ) or mongoose (*Urva auropunctata*). As we did not capture any reptilian frugivore activity through our camera traps and as nearly 83% of lizard frugivory marks were found on just Antigua and Grenada, we decided to focus more on mammalian and avian frugivory. In terms of frugivore activity, Nevis had the highest non‐native mammalian frugivore activity with 18 camera‐verified visits, while four islands (Antigua, Montserrat, Saba, and St. Eustacius) captured zero mammalian activity. The cameras also captured 12 instances of frugivorous avian activity, with Montserrat having the highest at seven verified visits, with all six species being native to the islands ( *Loxigilla noctis* (Lesser Antillean bullfinch), *Margarops fuscatus* (Pearly‐eyed thrasher), *Cinclocerthia ruficauda* (Brown trembler), *Elaenia martinica* (Caribbean elaenia), *Coereba flaveola* (Bananaquit), *Thamnophilus doliatus* (Barred Antshrike); Figure 2; Table S2).

**FIGURE 3:**
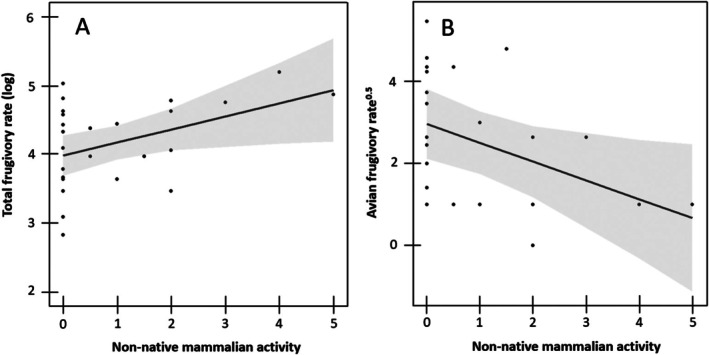
Relationship between non‐native mammalian frugivore activity and total frugivory rate (log‐transformed; *p* = 0.04; A) and native avian frugivory rate (square root transformed; *p* = 0.04; B).

We found a positive relationship between non‐native mammalian frugivore activity and total frugivory rate (*F* = 5.08, df = 12, *p* = 0.04; marginal *R* ^2^ = 0.18). We also found a negative relationship between avian frugivory rate (as measured by beak marks on the artificial fruits) and non‐native mammalian frugivore activity (*F* = 7.11, df = 12, *p* = 0.036; marginal *R* ^2^ = 0.16; Figure 3). Meanwhile, we did not find any significant correlations between non‐native mammalian frugivore activity and local avian community characteristics (abundance, species evenness, and richness) (*r*: 0.011 to 0.10; *p*: 0.69–0.97).

For predicting total frugivory rate, the model that included mammalian frugivore activity, avian frugivore activity, and site elevation best fit the data (*R* ^2^ = 0.72). Of the variables included in the best fitting model, mammalian frugivore activity (lmg = 0.58) had the highest relative contribution, compared to avian frugivore activity (lmg = 0.22) and site elevation (lmg = 0.2). From these variables, mammalian and avian frugivore activity were positively correlated with total frugivory rates, while site elevation was negatively correlated with total frugivory rates. No other model had a ΔAICc < 2 (Table 1).

**TABLE 1:** Best supported models (ΔAICc < 2) for predicting total frugivory rate (top) and non‐native mammalian frugivorous activity (bottom).

	Total frugivory rate (log)		Elevation	Df	ΔAICc	Weight	*R* ^2^
	Mammalian activity (sqrt)	Avian activity (sqrt)					
Model 1	+0.58	+0.22	−0.20	5	0	1	0.72

	Non‐native mammalian frugivore activity (sqrt)		Df	ΔAICc	Weight	*R* ^2^
	Distance to nearest island	Distance to nearest road (log)				
Model 1	−0.83	−0.17	4	0	0.72	0.64
Model 2	−1		3	1.87	0.28	0.42

*Note:* Relative importance (lmg scores) of each variable in the best supported models is included. Signs next to the relative importance values (“+” or “−”) indicate the direction of correlation. Weight comparisons were made only among this set of best supported models.

When investigating the factors that best predict non‐native mammal frugivore activity, the best‐supported model included distance to nearest island and distance to nearest road. A model that included just “distance to nearest island” had the next highest support (ΔAICc = 1.87). The best fitting model had an Akaike weight of 0.72 and an *R* ^2^ value of 0.6, while the next best‐supported model had an Akaike weight of 0.28 and an *R* ^2^ value of 0.4. From the best fitting model, distance to nearest island had the highest relative importance with a lmg score of 0.83, while distance to nearest road had a lmg score of 0.17. Both variables were negatively correlated with non‐native mammalian frugivore activity, indicating reduced non‐native mammalian activity on more isolated islands and at sites more distant from roads (Table 1). All variables within the best‐fitting models for either total frugivory rate or non‐native mammalian frugivore activity had VIF scores < 2, indicating low levels of multicollinearity.

## 4 Discussion

Across the eastern Caribbean islands, we found that non‐native mammals accounted for nearly 80% of all frugivore activity (frugivorous interactions identified by camera traps). Rodents (specifically *Rattus* spp.) were by far the most abundant non‐native mammals, with all but two of the 49 non‐native mammal visits being by rats. Moreover, non‐native mammalian frugivore activity was significantly and positively associated with total frugivory rates. Increased non‐native mammalian frugivore activity (as measured with our camera traps) was also associated with significant declines in avian frugivory rates (frugivorous interactions identified through plasticine fruit markings). This suggests that non‐native mammals suppress native frugivory interactions, as all birds detected by our camera traps were natives.

In all instances in which camera traps detected non‐native mammal interactions with the plasticine fruits, native frugivores were not detected at all, possibly due to non‐native mammals' tendency to remove every fruit. Therefore, it is possible that the suppressive effect is due to exploitative competition (Kawata 1997), in which non‐native mammals find and consume fruits before native frugivores. However, it could also be that non‐native mammals directly affect native avian abundance through predation or indirectly by altering their behavior. Every non‐native mammal species encountered in our study (rodents, dogs, and mongoose) are known to predate on birds, which in turn has been linked to behavioral changes and declines in population abundance of several bird species (Seaman and Randall 1962; Banks and Bryant 2007; Wilson Rankin et al. 2018). Since our analysis showed no association between relative avian abundance and diversity with increased non‐native mammal activity, one possible explanation is that differences in rodent density among islands influenced the frequency with which native frugivores encountered artificial fruits. Rather than directly suppressing native frugivores through predation, higher rodent densities could increase exploitative competition by consuming fruits earlier, thereby reducing fruit availability for native frugivores. Under this mechanism, areas with higher rodent densities would be expected to exhibit greater and earlier fruit consumption by rodents and fewer opportunities for native frugivores to interact with the artificial fruits. However, we cannot rule out the potential of changes in native frugivore behavior due to mammalian predator presence as another explanation for the negative relationship between non‐native mammalian frugivore activity and native avian frugivory rate. We also caution that the number of frugivores captured in the camera traps is a product of both their true occurrence and our ability to detect them, which is imperfect. Thus, a lack of frugivore detections (either native or non‐native) in camera traps does not necessarily suggest their total absence in an area.

The phenomenon of non‐native mammals suppressing native species interactions is likely amplified in areas such as many Caribbean islands where non‐native rats and other non‐native mammal populations are already abundant (Dalsgaard et al. 2007; Hays and Conant 2007; Goedert et al. 2020; Cooper 2008). While it is likely that a combination of exploitive competition and direct predation are contributing to the suppression of native species interactions, further studies are needed to measure the distinct contribution of each process in the eastern Caribbean. For instance, exclusion plot experiments, in which non‐native mammals are excluded from specific plants or plots to measure changes in frugivory rates, have been used by Case and Tarwater (2023) to assess the potential of exploitive competition. A similar approach could also be used to assess the role of direct predation on native frugivores by comparing population abundance, diversity, and behavior of native frugivore species in areas with and without non‐native mammals, as have been done by Wilson Rankin et al. (2018). However, if such a study is undertaken in areas that naturally have or lack non‐native mammals rather than through manipulating non‐native mammal abundances, underlying habitat variation that is associated with non‐native mammals' ability to reach and/or persist at the site could be the true driver of any observed differences rather than the non‐native mammals themselves. Our study is similarly observational rather than experimental, and the relatively small sample of 13 islands limits confidence in landscape scale associations. Habitat variation among islands, differences in non‐native mammals' ability to access sites, seasonality, and the variation in the abundance or detectability of native frugivores could all contribute to the observed relationships between mammal activity and geographic variables. Consequently, these correlations should not be interpreted as evidence of causal relationships. Future studies that experimentally manipulate non‐native mammalian frugivore abundance, include a larger number of islands spanning broader environmental gradients, and monitor frugivore communities over multiple seasons would help disentangle these potentially confounding factors and determine the consistency of the observed landscape scale patterns.

Beyond the negative correlation we observed between non‐native mammalian frugivore activity and native avian frugivory, our model selection results further suggest a strong role for non‐native mammals in shaping plant‐frugivore dynamics in this region. We found that total frugivory rate (as detected through removed or marked plasticine fruits) was best predicted by the number of non‐native mammals detected by the camera traps, and that the relative importance of this variable was greater than all other identified variables (bird activity and elevation) combined (Table 1). This suggests that non‐native mammals are currently dominating frugivory dynamics in the region. Such dominance has deleterious implications for native frugivory interactions. Almost all non‐native mammalian frugivore activity found in our study was from non‐native rodents, which are known to negatively affect plant communities as rodents mostly predate seeds rather than performing seed dispersal (Miller‐ter Kuile et al. 2021; Galetti et al. 2015). If rodents are indeed the dominant frugivores in these ecosystems, this bodes poorly for the fitness of many plant communities and the native animal species that associate with them. For instance, Shiels and Drake (2015) found that invasive rats had a devastating effect on native palm trees in Hawaii by dramatically increasing seed mortality rates. Invasive rodents were also linked with reduced recruitment capacity of trees on Lord Howe Island (Auld et al. 2010). Meanwhile, Case and Tarwater (2023) found that the presence of invasive rodents led to declines in frugivory levels, which in turn had direct (via seed destruction) and indirect (via reduction in frugivory rates) effects on plant fitness by reducing seed dispersal and recruitment success. While smaller seeds may survive rodent consumption, non‐native mammals remain unreliable substitutes for native frugivores as the minimal benefits they may provide are heavily outweighed by the damage they cause (Shiels et al. 2020). Given these findings and the high levels of non‐native rodent frugivory documented in our study, it is likely that plant communities in the eastern Caribbean islands also suffer from decreased seed‐dispersal potential and lower reproduction rates.

The results of our model selection for predicting non‐native mammalian frugivore activity highlight two main factors. First, our top model found an association between distance to roads and mammal activity, with higher activity being found closer to roads. Roads are commonly associated with areas of high human activity (Matlack 1993) and have been linked to higher abundances of non‐native species (McDonald and Urban 2006). However, an island‐level isolation metric—distance to the nearest neighboring island—had the higher predictive effect by far, with islands that had closer neighbors being associated with higher non‐native mammalian frugivore activity. While initially this result may not be surprising, given that less isolated areas are associated with higher economic connectivity (Gleditsch et al. 2022) and thus possess more pathways for non‐native species introductions (Chapman et al. 2017), it is noteworthy that this biogeographic effect had a higher predictive strength than the considered socio‐economic variables. One potential reason for this finding may be that the socio‐economic variables used for this study represent current socio‐economic characteristics and may not be representative of historic socio‐economic trends when non‐native mammals were likely first introduced. Additionally, we were unable to consider shipping levels for this study, despite many non‐native animals being known to be transported through these means, as historic records of shipping for many of the islands in the eastern Caribbean are largely lacking. This potential driver of non‐native mammalian frugivore activity may thus be worth investigating in future studies. Regardless, our results highlight the important role that an island's overall biogeographic connectivity may have in shaping non‐native mammalian frugivore activity. It is also worth noting that none of the variables strongly correlated with island area (distance to build areas, native plant richness, length of roads, and number of tourists) were included in the top models, which emphasizes the relative importance of island connectivity over island size in predicting non‐native mammalian frugivore activity.

In conclusion, our results support previous studies' assertion that non‐native mammals are disrupting native frugivory dynamics on islands (Heinen et al. 2023), potentially to a level where they are negatively influencing seed‐dispersal and plant reproduction, notably by exploiting fruit resources more quickly than native frugivores. Furthermore, we link island connectivity and proximity to anthropogenic infrastructure with higher non‐native mammalian frugivore activity, showing that both island biogeography and human activity can indirectly affect frugivory in the eastern Caribbean by affecting invasive species dynamics. Given the generally harmful and seed predatory behavior of introduced rats and many other non‐native mammals (Miller‐ter Kuile et al. 2021), our study suggests that populations of non‐native mammals—especially rodents—should be controlled not only to protect island animal populations (Blackburn et al. 2004), but also to ensure the reproduction (seed‐dispersal) and long‐term survival of island plant communities and healthy ecosystem functioning. Complete eradication of invasive rodents has been successfully carried out, particularly in small and isolated ecosystems such as islands (Howald et al. 2007). However, eradication efforts on tropical islands have historically been difficult due to factors such as non‐target rodenticide consumption and ongoing agricultural activity, which may provide invasive rodents with an alternative food source that decreases interest in bait (Holmes et al. 2015). Consequently, it may be more practical to prioritize rodent control in ecologically sensitive areas or sites of high conservation value in the Lesser Antilles rather than attempting to remove them from entire islands in this region (Jones et al. 2016; Holmes et al. 2019).

## Author Contributions

**Seokmin Kim:** conceptualization (lead), data curation (lead), formal analysis (lead), investigation (equal), methodology (equal), project administration (lead), software (lead), validation (lead), visualization (lead), writing – original draft (lead), writing – review and editing (lead). **Fabio L. Tarazona‐Tubens:** conceptualization (supporting), data curation (supporting), investigation (equal), methodology (equal), writing – review and editing (supporting). **Maximilian G. R. Vollstädt:** conceptualization (supporting), investigation (supporting), methodology (supporting), supervision (supporting), validation (supporting), writing – review and editing (supporting). **Fernando Gonçalves:** conceptualization (supporting), investigation (supporting), methodology (supporting), supervision (supporting), validation (supporting), writing – review and editing (supporting). **Emmeli Agerskov Claré:** investigation (supporting), methodology (supporting). **Andreas Krogh Norrild:** investigation (supporting), methodology (supporting). **Hanna Welzel:** investigation (supporting), methodology (supporting). **Tianying Zhang:** investigation (supporting), methodology (supporting). **Mark Hulme:** supervision (supporting), writing – review and editing (supporting). **Christopher Kaiser‐Bunbury:** supervision (supporting), supervision (supporting), writing – review and editing (supporting), writing – review and editing (supporting). **Mauro Galetti:** conceptualization (supporting), supervision (supporting). **Benno Simmons:** supervision (supporting), writing – review and editing (supporting). **Bo Dalsgaard:** conceptualization (supporting), funding acquisition (lead), project administration (supporting), resources (supporting), supervision (equal), writing – review and editing (supporting). **Christopher Searcy:** conceptualization (equal), project administration (equal), resources (equal), supervision (equal), writing – original draft (supporting), writing – review and editing (supporting).

## Funding

This work was supported by the Danmarks Frie Forskningsfond (0135‐00333B), the Marie Curie Postdoctoral Fellowship (Horizon‐TMA‐MSCA‐101149502), the María de Maeztu Project CEX2021‐001198‐M to IMEDEA (CSIC‐UIB) and the Swiss National Science Foundation Postdoctoral fellowship (TMPFP2_217531).

## Conflicts of Interest

The authors declare no conflicts of interest.

## Supporting information


**Table S1:** List of variables considered for model selection, transformations made to optimize normality, predictor category, and those included in the model selection after removing highly correlated variables.
**Figure S1:** Characteristic marks of birds (1–4) and reptiles (5–6) on artificial fruits.

**Table S2:** Full data used for our analyses.

## Data Availability

All data used in this paper and relevant supporting materials are included as Supporting Information S1.
