# Supplementary material for: Non‐Native Rodents Dominate Understory Frugivory in the Eastern Caribbean Islands
Source: Ecol Evol. 2026 Jul 31;16(8):e74116. doi: 10.1002/ece3.74116 (PMC13426735; doi:10.1002/ece3.74116)
Supplement: Supplementary file 1 — Table S1: List of variables considered for model selection, transformations made to optimize normality, predictor category, and those included in the model selection after removing highly correlated variables. Figure S1: Characteristic marks of birds (1–4) and reptiles (5–6) on artificial fruits. [file ECE3-16-e74116-s002.docx]

**SUPPLEMENTARY INFORMATION**

**Data availability statement**

All data used in this paper and relevant supporting materials are available through GitHub (https://github.com/sxk1332/artificial_fruits).

| Variable | Transformation | Category | Selected |
| --- | --- | --- | --- |
| Latitude | - | Biogeographic |  |
| Distance to continent | - | Biogeographic |  |
| Number of neighbors | - | Biogeographic | X |
| Island area | log | Biogeographic |  |
| Island age | log | Biogeographic |  |
| Distance to nearest neighbor | - | Biogeographic | X |
| Elevation range | - | Biogeographic |  |
| Mass effect | log | Biogeographic |  |
| Reptile richness | - | Biotic | X |
| Percent forest cover | - | Biotic | X |
| Bird richness | inverse | Biotic | X |
| Native plant richness | - | Biotic | X |
| Site elevation | - | Local | X |
| Mammal visits | sqrt | Local | X |
| Distance to nearest road | log | Local | X |
| Distance to nearest built area | log | Local |  |
| Bird visits | sqrt | Local | X |
| Percent built cover | - | Socio-economic | X |
| Percent crop cover | log | Socio-economic |  |
| GDP per capita | log | Socio-economic | X |
| Total road length | log | Socio-economic |  |
| Number of tourists | log | Socio-economic | X |

**Table S1**: List of variables considered for model selection, transformations made to optimize normality, predictor category, and those included in the model selection after removing highly correlated variables.

**Table S2:** Full data used for our analyses.


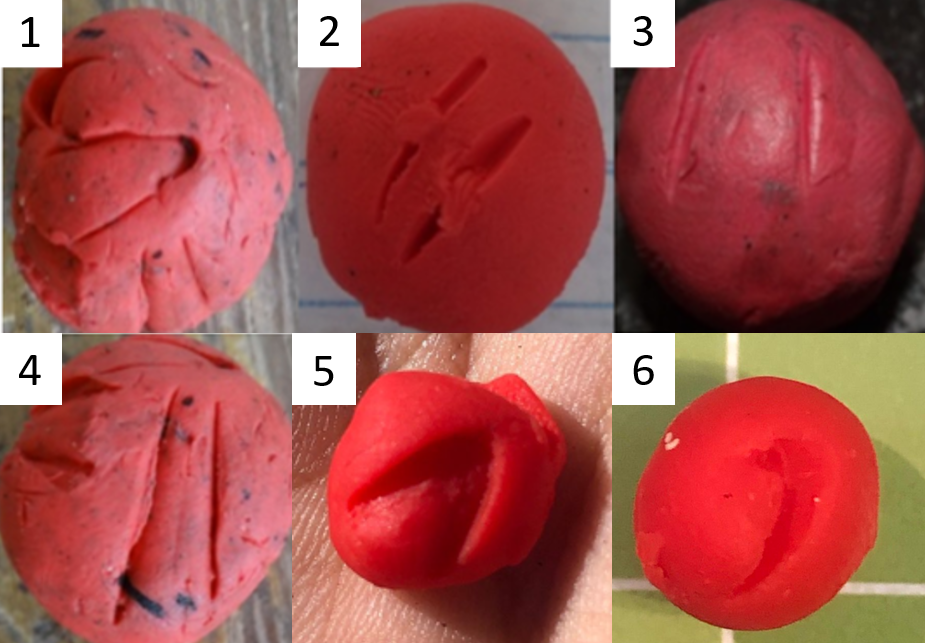


**Figure S1**: Characteristic marks of birds (1-4) and reptiles (5-6) on artificial fruits.
